# Antifungal Mechanism and Efficacy of Kojic Acid for the Control of *Sclerotinia sclerotiorum* in Soybean

**DOI:** 10.3389/fpls.2022.845698

**Published:** 2022-03-11

**Authors:** Gui-Yang Zhu, Xin-Chi Shi, Su-Yan Wang, Bo Wang, Pedro Laborda

**Affiliations:** ^1^School of Life Sciences, Nantong University, Nantong, China; ^2^Xuzhou Institute of Agricultural Sciences in Xuhuai District, Sweet Potato Research Institute, Xuzhou, China

**Keywords:** kojic acid, *Sclerotinia sclerotiorum*, antifungal activity, melanin biosynthesis, soybean pathogens

## Abstract

Sclerotinia stem rot, which is caused by the fungal pathogen *Sclerotinia sclerotiorum*, is a soybean disease that results in enormous economic losses worldwide. The control of *S. sclerotiorum* is a difficult task due to the pathogen’s wide host range and its persistent structures, called sclerotia. In addition, there is lack of soybean cultivars with medium to high levels of resistance to *S. sclerotiorum*. In this work, kojic acid (KA), a natural bioactive compound commonly used in cosmetic industry, was evaluated for the management of Sclerotinia stem rot. Interestingly, KA showed strong antifungal activity against *S. sclerotiorum* by inhibiting chitin and melanin syntheses and, subsequently, sclerotia formation. The antifungal activity of KA was not obviously affected by pH, but was reduced in the presence of metal ions. Treatment with KA reduced the content of virulence factor oxalic acid in *S. sclerotiorum* secretions. Preventive applications of 50 mM KA (7.1 mg/ml) completely inhibited *S. sclerotiorum* symptoms in soybean; whereas, in curative applications, the combination of KA with prochloraz and carbendazim improved the efficacy of these commercial fungicides. Taken together, the antifungal activity of KA against *S. sclerotiorum* was studied for the first time, revealing new insights on the potential application of KA for the control of Sclerotinia stem rot in soybean.

## Introduction

Soybean, *Glycine max* (Fabaceae: Phaseoleae), is extensively cultivated in China, United States, Brazil, and Argentina (Ding et al., 2021; Hu et al., 2021). The global soybean production in 2017 was 359.5 million tons; however, soybean production declined to 333.7 million tons in 2019 [Food and Agricultural Organization of the United Nations (FAO), 2019]. This decrease has been mainly attributed to yield losses produced by soybean diseases, such as Sclerotinia stem rot, which is caused by *Sclerotinia sclerotiorum* (Willbur et al., 2018; Zhang et al., 2019). *Sclerotinia sclerotiorum* is known to adhere to the host surface and to penetrate the plant cuticle (Liu et al., 2018; Xu et al., 2018). This fungal pathogen is able to invade different soybean tissues, such as stem, root, and pod, at different plant growth stages (Gao et al., 2020). It was estimated that the losses produced by *S. sclerotiorum* in soybean in 1997, 2004, and 2009 were approximately in 953 million, 1.63, and 1.61 billion kg, which correspond to 227, 344, and 560 million dollars, respectively (Peltier et al., 2012).

Currently, there is lack of soybean cultivars with medium to high levels of resistance to *S. sclerotiorum*, and crop rotation is not fully effective for the control of this pathogen (Gracia-Garza et al., 2002; Ranjan et al., 2019). At present, the management of *S. sclerotiorum* in soybean strongly relies on the use of chemical fungicides, such as carbendazim, fluazinam, prochloraz, and procymidone (Miorini et al., 2021). However, none of them offer complete control and several resistant strains have been reported (Mao et al., 2020; Zhou et al., 2020). Apart from the low efficacy of commercial fungicides, the management of *S. sclerotiorum* is a difficult task due to the pathogen’s wide host range, and its persistent resting structures, called sclerotia. These are known to play important roles in fungal life and disease development cycles (Xia et al., 2020; Tian et al., 2021). Sclerotia can produce apothecia and release spores, which can be transmitted by wind and rain, to infect plants under appropriate temperature and humidity conditions (Clarkson et al., 2014; Becka et al., 2016). Sclerotia can also germinate and directly infect the host plant. Moreover, sclerotial exudates have been reported to help in the development of sclerotia, and facilitate host cell necrosis (Bolton et al., 2006). The process of mycelial germination from sclerotia is controlled by melanin, which induces resistance to adverse environmental conditions (Huang, 1991). The melanin in *S. sclerotiorum* is mainly composed by 1,8-dihydroxynaphthalene (DHN) and is deposited in peripheral rind cells during sclerotia formation (Butler et al., 2009; Eisenman and Casadevall, 2012; Ordonez-Valencia et al., 2015).

In order to develop efficient methods for the management of Sclerotinia stem rot, several research groups have developed biocontrol strategies using *Bacillus* spp., and have achieved generally low–moderate control efficacies (46–76% inhibition rate; Radhakrishnan et al., 2017; Lopes et al., 2018; Massawe et al., 2018). Among the biocontrol strains *Coniothyrium minitans* CON/M/91–08, *Streptomyces lydicus* WYEC 108, *Trichoderma harzianum* T-22, and *Bacillus subtilis* QST 713 screened for the control of Sclerotinia stem rot, the highest biocontrol efficacy was obtained when using *C. minitans* (68.5% disease severity inhibition; Zeng et al., 2012; Zhao et al., 2020). Similarly, *Clonostachys rosea*, *Stachybotrys levispora*, *Trichoderma asperelloides*, and *Sporidesmiun sclerotivorum* have also been evaluated for the management of *S. sclerotiorum* in soybean, and in this case, *S. sclerotivorum* reduced Sclerotinia stem rot by 56–100% in commercial fields (Del Rio et al., 2002; Rodriguez et al., 2011; Ribeiro et al., 2018; Sumida et al., 2018). Wang et al. (2019) discovered two mycoviruses in *S. sclerotiorum* SZ-150, while *Bacillus amyloliquefaciens* VB7 was reported to inhibit *S. sclerotiorum* mycelial growth by 45% and sclerotial production by 100% (Vinodkumar et al., 2017). The volatiles produced by *Pseudomonas brassicacearum*, *brassicacearum putida*, and *Bacillus megaterium* altered the cytoplasm and organelle organization in *S. sclerotiorum* (Giorgio et al., 2015).

On the other hand, kojic acid (KA, 5-hydroxy-2-hydroxymethyl-γ-pyrone) is a common secondary metabolite produced by numerous fungal strains and is widely used in cosmetic industry (Saeedi et al., 2019; Song et al., 2021). The accumulation of tyrosinase in human cells involves the biosynthesis of melanin, leading to skin hyperpigmentation. Tyrosinase contains copper ion in the active site, and KA forms a complex with it thereby reducing enzymatic activity. For this reason, KA is used as a skin lightener, protecting the skin from the UV radiation (Cabanes et al., 1994). KA has been also used as a food additive to preserve postharvest fruit and vegetables from browning (Cabezudo et al., 2021). The antioxidant and chelating properties of KA, together with its natural safety, non-significant environmental side effects, and the ability for large-scale production, have stimulated the research on the availability and possible applications of KA during recent years, with 179 articles published in 2020. Although the ability of KA to inhibit melanin biosynthesis in human cells suggests that KA may be able to inhibit melanin biosynthesis and sclerotia formation in *S. sclerotiorum*, the potential application of KA for the control of plant pathogens *in vivo* remains completely unexplored. In this work, the antifungal properties of KA were evaluated as a new low cost alternative for *S. sclerotiorum* disease management.

## Materials and Methods

### Fungal Strains

*Sclerotinia sclerotiorum* strain NJC09 was isolated from soybean plants showing Sclerotinia stem rot symptoms in Xuzhou, China (117.30° E, 34.28° N). Isolation and identification of the pathogen are described in Supplementary Figure S1. The isolation of *Valsa pyri* strain Vp297, *Colletotrichum brevisporum* strain GM1, and *Botryosphaeria dothidea* isolate SBL303 was previously reported by our research group (Chen et al., 2020; Shi et al., 2020; Song et al., 2020). Fungal strains were cultured on potato–dextrose–agar (PDA) medium (200 g potato, 20 g dextrose, and 15 g agar in 1 L water) at 28°C.

### Antifungal Activity Assay

Fungal plant pathogens *B. dothidea*, *C. brevisporum*, *S. sclerotiorum*, and *V. pyri* were used in the antifungal assay. KA was purchased from Macklin (China). The antifungal activity of KA was evaluated in PDA medium containing 5, 10, and 15 mM KA (0.71, 1.42, and 2.13 mg/ml KA), respectively. The control experiments were performed in the absence of KA. The cultures were incubated under darkness at 28°C for 3 days. The colony diameter was measured using a ruler (Tremarin et al., 2015), and the antifungal effect was determined according to the mycelial growth. Experiments were repeated five times.

To calculate the half maximal effective concentrations (EC_50_), KA, carbendazim, and prochloraz were used. *S. sclerotiorum* was grown on PDA plates containing 0.5, 1, 2, 3, 5, 10, and 15 mM of each fungicide (KA: 0.071, 0.14, 0.28, 0.43, 0.71, 1.42, and 2.13 mg/ml; carbendazim: 0.096, 0.19, 0.38, 0.57, 0.96, 1.91, and 2.87 mg/ml; prochloraz: 0.19, 0.38, 0.75, 1.13, 1.88, 3.77, and 5.65 mg/ml). The negative control experiment was performed by culturing *S. sclerotiorum* on PDA in the absence of fungicides. The dishes were incubated at 28°C for 3 days. Experiments were repeated five times. Prism 7.0 (GraphPad Software, United States) was used to calculate the EC_50_ values.

### Effect of pH and Metals on the Inhibitory Activity of KA

*Sclerotinia sclerotiorum* was cultured on PDA medium under darkness at 28°C for 5 days. Three plugs (7 mm diameter) of mycelium were inoculated into 40 ml honey–barley–tryptone medium (600 mg honey, 100 mg barley, and 200 mg tryptone in 40 ml water) and placed at 28°C and 200 rpm for 12 h. After centrifugation at 8,000 rpm and 4°C, the produced mycelial fragments were washed twice with 20 ml sterilized distilled deionized water (ddH_2_O). Harvested cells were suspended in 200 μl yeast extract–peptone–dextrose medium (YEPD; 0.15 g yeast extract, 0.5 g peptone, and 1 g glucose in 50 ml water; 1 × 10^6^ mycelial fragments/ml) containing 0 and 10 mM KA (0 and 1.42 mg/ml KA). The number of mycelial fragments was calculated using a Leica DM2500 microscope (Germany) at ×40 magnifications and adjusted using sterilized ddH_2_O.

The effect of pH was calculated by adjusting the YEPD medium to pH values 3, 4, 5, 6, 7, and 8. The effect of iron(II), nickel(II), zinc(II), cobalt(II), and copper(II) was examined by adding the corresponding metal chlorides (1 mM final concentration) into the YEPD medium (pH 4). After 12 h of culture at 28°C and 200 rpm, fungal growth was detected using a Leica DM2500 microscope at ×20 magnifications. The antifungal activity was calculated according to the number of sclerotial aggregates per microliter. Experiments were repeated three times.

### Fluorescent Live-Cell Imaging

*Sclerotinia sclerotiorum* mycelial fragments were cultured in YEPD medium with 0 and 10 mM KA (0 and 1.42 mg/ml KA) as described in “Effect of pH and Metals on the Inhibitory Activity of KA” section. After 12 h at 28°C and 200 rpm, scanning electron microscope (SEM Gemini 300 Instrument, United States) and fluorescent images (Olympus BX51, Japan) were acquired. For the preparation of SEM samples, fungal cells were centrifuged and immobilized in 4% paraformaldehyde. The samples were dehydrated with 65, 75, 85, 95, and 100% (twice) ethanol at room temperature (each dehydration condition was carried out for 10 min). Then, the samples were stored in a desiccator for 6 h. Dried samples were placed on the sample holder, and coated with gold. Images were obtained with an acceleration voltage of 20 kV. In the analysis using the fluorescent microscope, 10 μl YEPD medium was stained with 10 μl 4,6-diamino-2-phenylindole (DAPI, 10 μg/ml solution, Solarbio, China). The resulting suspension was kept in darkness during 5 min before the analysis. Images were collected using a DAPI-selective filter (excitation wavelength: 377 nm; emission wavelength: 447) at ×40 magnifications.

### Determination of DHN Content

Two 7-mm-diameter PDA plugs containing *S. sclerotiorum* mycelium were inoculated into 50 ml YEPD medium with 0, 2, 5, and 10 mM KA (0, 0.28, 0.71, and 1.42 mg/ml KA). After 15 days at 28°C and 200 rpm, DHN was extracted following the standard procedure (Kogej et al., 2004) and DHN content was determined by analytical HPLC-MS using a QTRAP 5500 system (AB SCIEX, United States), equipped with an Agilent C18 column (250 × 4.6 mm, United States). The system was maintained at 30°C. The mobile phase was 0.1% formic acid aqueous solution/acetonitrile 30:70. The injection volume was 5 μl and the flow rate was 0.2 ml/min. The peak corresponding to DHN was detected at 1.26 min. The concentration of DHN was calculated according to the peak area in MS using the transition from 161.0 to 143.3 [(M + H)^+^ calcd. For C_10_H_9_O_2_, 161.0603; found, 161.0]. Experiments were repeated three times.

### Detection of mRNA Levels (qRT-PCR) in *Sclerotinia sclerotiorum*

Freshly harvested cells prepared as described above were resuspended in YEPD medium (8 ml, 1 × 10^6^ mycelial fragments/ml) with 0 and 10 mM KA (0 and 1.42 mg/ml KA), and shake at 28°C and 200 rpm for 24 h. After centrifugation at 8,000 rpm and 4°C, the formed aggregates were collected. Total RNA was extracted using TRIzol reagent (Ambion, United States). cDNA was obtained by reverse transcription using the Transcript All-in-One First-Strand DNA Synthesis SuperMix for qPCR (OneStep gDNA Removal) Kit (Tsingke, China). qRT-PCR was performed using a set of two PCR primers with SYBR Green I Real-Time PCR (Solarbio, China) using a 7500 Real-time PCR system (Applied Biosystems, United States). Genes related to cell wall formation (*CHS1*, *CHS2*, *CHS3*, and *GSH*) and melanin synthesis (*PKS12* and *PKS13*) were examined. *Actin* transcript was used as the internal reference gene (Melo et al., 2019). Primers are shown in Supplementary Table S1. The relative gene expression was calculated by the 2^−ΔΔCt^ method. Experiments were repeated five times.

### Determination of Chitin Content

After suspension of *S. sclerotiorum* mycelial fragments in 50 ml YEPD medium (1 × 10^6^ mycelial fragments/mL) containing 0 and 10 mM KA (0 and 1.42 mg/ml KA), the cells were shaken at 28°C and 200 rpm for 0, 1, 2, and 3. Chitin content was determined using the procedure described by Song et al. (2020). Briefly, after centrifugation (8,000 rpm, 4°C, 10 min) of the cells contained in the 50 ml YEPD medium, the fungal cells were hydrolyzed in 1 ml 6 M HCl at 100°C for 17 h. After evaporation of the solvent using a freeze-drier (FreeZone 2.5 Plus, LABCONCO, United States), the samples were dissolved in 1 ml water. Then, 0.75 M Na_2_CO_3_ solution (100 μl) was added to 100 μl sample. The mixture was incubated at 100°C for 20 min. Then, 95% ethanol (0.7 ml) and solution A (100 μl; solution A: 1.6 g *p*-dimethylaminobenzaldehyde in 30 ml concentrated HCl and 30 ml ethanol) were added. The absorbance was measured at 420 nm and compared with the standard curve from 0.1 to 20 mg/ml glucosamine. Experiments were repeated three times.

### Determination of Oxalic Acid Content

Two 7-mm-diameter PDA plugs containing *S. sclerotiorum* mycelium were inoculated into 50 ml YEPD medium with 0, 2, 5, and 10 mM KA (0, 0.28, 0.71, and 1.42 mg/ml KA). Samples were collected after 0, 1, 2, and 3 days. The content of oxalic acid was measured using the Oxalic Acid Content Detection (Visible Spectrophotometry) Kit (Solarbio, China). Briefly, 50 μl of culture medium were mixed with reagents 1, 2, and 3 following the manufacturer’s instructions, and the resulting solution was kept at room temperature for 20 min. The concentration of oxalic acid was calculated according to the absorbance at 510 nm. Experiments were repeated three times.

### Curative and Preventive Efficacies of KA for the Management of *Sclerotinia sclerotiorum* in Soybean Pods

*Sclerotinia sclerotiorum* was cultured on PDA medium at 28°C for 5 days, and the mycelium was divided into 7-mm-diameter plugs. For the preventive assay, 2-mm-diameter wounds were made on the surface of soybean pods directly detached from soybean plants. After spraying 50 ml aqueous solutions (pH 5) containing 0, 10, 20, and 50 mM KA (0, 1.42, 2.84, and 7.11 mg/ml KA), the soybean pods were dried, and a mycelial plug was kept in contact with the wound. Carbendazim and prochloraz at 20 and 50 mM (carbendazim: 3.82 and 9.56 mg/ml; prochloraz: 7.53, and 18.84 mg/ml) were applied, instead of KA, as positive controls (4 kg/ha carbendazim and 0.45 kg/ha prochloraz are the recommended rates in fields trials). After 3 days at 28°C and 70% humidity, the preventive efficacy was measured according to the lesion length (necrotic area) caused by *S. sclerotiorum*.

For the curative assay, 2-mm-diameter wounds were made on the soybean pods. After inoculation using a mycelial plug, the inoculated soybean pods were incubated at 28°C and 70% humidity for 1 day and the mycelial plugs were removed. Then, 50 ml aqueous solutions containing KA, prochloraz, or carbendazim at 20 and 50 mM concentration were sprayed. Sterilized ddH_2_O (50 ml) was sprayed in the control experiment. Treatments with combinations of fungicides were carried out using a 50 ml solution containing the fungicides at 10 mM concentration (KA: 1.42 mg/ml; carbendazim: 1.91 mg/ml; and prochloraz: 3.77 mg/ml).

Twenty soybean pods were used for each treatment condition. Experiments were repeated three times. The brownish-black color of the lesions caused by *S. sclerotiorum*, covered in some cases by white hyphae, was easily observed with the naked eye. All the lesion length measurements were pooled to calculate the mean value and deviation.

### Data Analysis

The statistical analyses were performed using SPSS (Statistical Package, Version 20.0). The variables were subjected to student’s *t*-test and were tested for significance at *p* < 0.05 (*), *p* < 0.01 (**), *p* < 0.001 (***), and *p* < 0.0001 (****) levels (ns = no significance). The SD, which was calculated using Microsoft Excel 2010, was used to quantify the dispersion.

## Results

### KA Shows Antifungal Activity Against *Sclerotinia sclerotiorum* and Inhibits Sclerotia Formation

**Figure 1:**
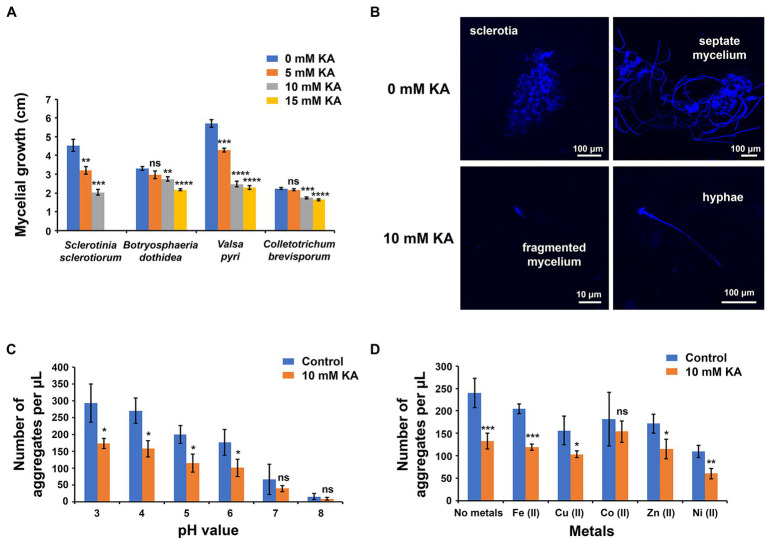
Antifungal properties of kojic acid (KA). **(A)** Antifungal activity of KA against fungal pathogens *Sclerotinia sclerotiorum*, *Botryosphaeria dothidea*, *Valsa pyri*, and *Colletotrichum brevisporum*. The antifungal activity was measured according to the inhibition of mycelial growth (each treatment mean represents the average of five repetitions). **(B)** Fluorescent microscope observations of *S. sclerotiorum* growth with and without KA treatment (10 mM; 1.42 mg/ml). **(C)** Effect of pH on the antifungal activity of 10 mM KA against *S. sclerotiorum* (each treatment mean represents the average of three repetitions). **(D)** Effect of metal ions (1 mM) on the antifungal activity of 10 mM KA against *S. sclerotiorum* at pH 4 (each treatment mean represents the average of three repetitions). Significance levels at ^*^p < 0.05, ^**^p < 0.01, ^***^p < 0.001, ^****^p < 0.0001, and no significance (ns). Bars represent the SD.

Although KA at 15 mM slightly reduced the mycelial growth of *B. dothidea*, *C. brevisporum*, and *V. pyri*, this concentration of KA inhibited completely the growth of *S. sclerotiorum* (Figure 1A), whereas 5 and 10 mM KA reduced *S. sclerotiorum* growth by 29 and 56%, respectively (Supplementary Figure S2). These results indicated that *S. sclerotiorum* is more sensitive to KA in comparison to the other fungal species used in the assay. After 15 days of incubation, no mycelial growth was detected when treating *S. sclerotiorum* with 15 mM KA. When comparing the antifungal activity of KA with that of commercial fungicides carbendazim and prochloraz, it was found that the commercial fungicides showed higher antifungal activity *in vitro* than KA. The EC_50_ value for KA was 6.3 ± 0.7 mM (0.89 ± 0.09 mg/ml), while the EC_50_ values for carbendazim and prochloraz were 2.8 ± 0.3 (0.51 ± 0.06 mg/ml) and 0.7 ± 0.2 mM (0.26 ± 0.07 mg/ml), respectively.

Fluorescent microscope observations revealed that, in the absence of KA, *S. sclerotiorum* mycelial fragments formed sclerotial aggregates (number of observed sclerotia: 64), and only a few non-attached hyphae were detected (number of observed hyphae: 26; Figure 1B; Supplementary Figure S3). In some cases, hyphal structures were observed growing from the sclerotial aggregates (number of observed sclerotia with hyphae: 53; number of sclerotia without hyphae: 11). In contrast, after treatment with 10 mM KA, most *S. sclerotiorum* mycelial fragments remained in the original state (number of observed mycelial fragments: 112), and the number of hyphae increased in comparison with that observed in the absence of KA (number of observed hyphae: 53). After treatment with KA, no sclerotial aggregates were observed, indicating that KA was able to inhibit the formation of sclerotia and, subsequently, to temporarily halt *S. sclerotiorum* cell cycle.

The antifungal activity of KA was screened at different pH values, from 3 to 8. Although *S. sclerotiorum* could grow at all pH values, the formation of sclerotial aggregates was higher in an acidic environment than under alkaline conditions. The number of aggregates per microliter was 270 at pH 3, whereas the number of aggregates decreased to 15 at pH 8. In all tested pH values, the inhibitory activity of KA against *S. sclerotiorum* varied from 40 to 45%, revealing that the antifungal activity of KA was not obviously affected by pH (Figure 1C).

The presence of metals inhibited the formation of sclerotial aggregates (Figure 1D). In this sense, nickel(II) and copper(II) reduced the number of aggregates by 53 and 32%, respectively, in comparison to the control experiment. When KA treatment was combined with metal ions, the antifungal effect of KA was significantly reduced, with cobalt(II) and zinc(II) producing the greatest effect. KA, in the presence of cobalt(II) and zinc(II), reduced the number of aggregates per μl by 28 and 33%, respectively; whereas KA, without metal addition, reduced the number of aggregates by 45%.

### KA Inhibits Melanin Biosynthesis in *Sclerotinia sclerotiorum*

**Figure 2:**
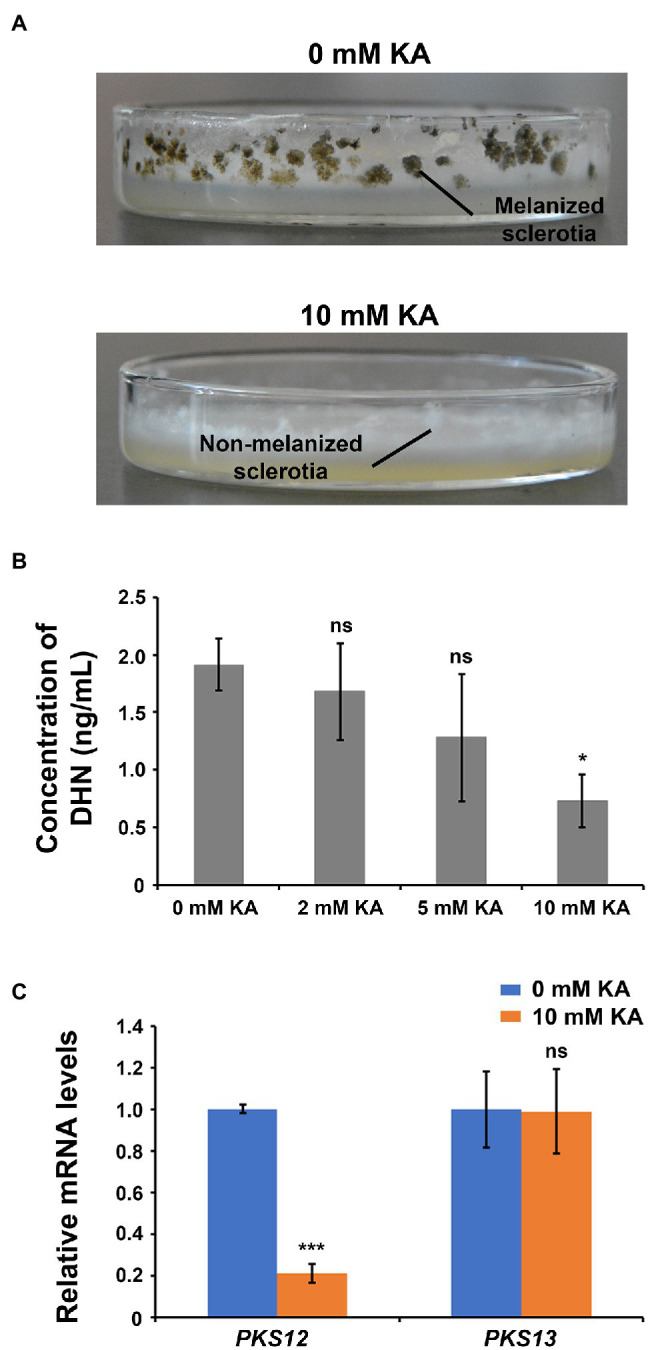
Kojic acid inhibits melanin biosynthesis and sclerotia formation in *Sclerotinia sclerotiorum*. **(A)** Images showing sclerotia formation after treatment with 10 mM KA (1.42 mg/ml KA). **(B)** Concentration of 1,8-dihydroxynaphthalene (DHN) in *S. sclerotiorum* cells after treatment with 10 mM KA (each treatment mean represents the average of three repetitions). **(C)** Relative mRNA levels of key genes involved in the biosynthesis of DHN and other melanin compounds after treatment with 10 mM KA (each treatment mean represents the average of five repetitions). Significance levels at ^*^p < 0.05, ^***^p < 0.001, and no significance (ns). Bars represent the SD.

Cultivation of *S. sclerotiorum* on PDA plates allowed the formation of sclerotia after 15 days (96 ± 19 sclerotia per plate), which could be easily observed due to the dark color produced by melanin (Figure 2A; Sousa Melo et al., 2019). Instead, the presence of 10 mM KA inhibited the formation of sclerotia (6 ± 3 sclerotia per plate), and cultures were completely white showing only non-melanized structures.

In order to confirm that KA was inhibiting the biosynthesis of melanin in *S. sclerotiorum*, the content of DHN, a kind of melanin and a key intermediate involved in biosynthesis of other melanin compounds in *S. sclerotiorum* (Supplementary Figure S4; Butler et al., 2009), was examined after application of different concentrations of KA. In agreement with the lack of dark sclerotia in the plates, 2, 5, and 10 mM KA reduced DHN content by 12% (no significant difference), 32% (no significant difference), and 62% (significant difference), respectively (Figure 2B); confirming that KA is inhibiting the biosynthesis of melanin. Consistently, the expression of polyketide synthase *PKS12*, which is involved in melanin biosynthesis, was found 4.8-fold downregulated in the presence of 10 mM KA, whereas no significant difference was observed in the mRNA level of polyketide synthase *PKS13* after KA treatment (Figure 2C).

### KA Inhibits Chitin Biosynthesis and Alters Cell Wall Integrity in *Sclerotinia sclerotiorum*

**Figure 3:**
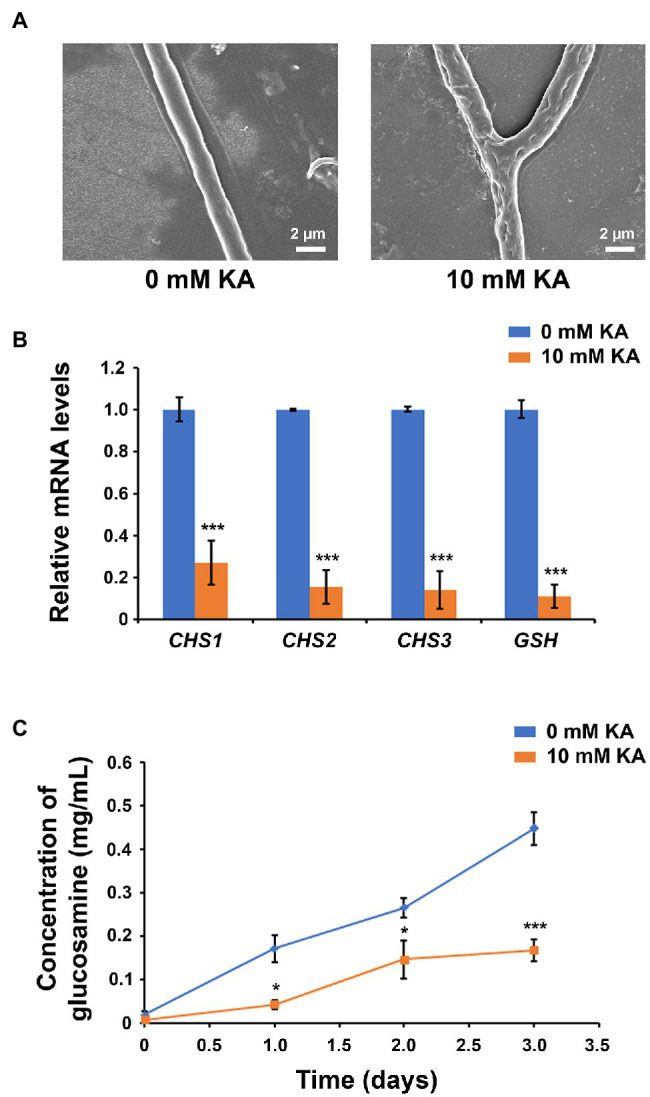
Kojic acid inhibits chitin synthesis and alters cell wall integrity in *Sclerotinia sclerotiorum*. **(A)** Scanning electron microscope (SEM) observations of *S. sclerotiorum* cell wall after treatment with 10 mM KA (1.42 mg/ml KA). **(B)** Relative mRNA levels of chitin synthase genes *CHS1*, *CHS2*, and *CHS3*, and β-1,3-glucan synthase gene *GSH*, in *S. sclerotiorum* after treatment with 10 mM KA (each treatment mean represents the average of five repetitions). **(C)** Concentration of chitin, measured by the concentration of glucosamine after hydrolysis, in *S. sclerotiorum* cells after treatment with 10 mM KA (each treatment mean represents the average of three repetitions). Significance levels at ^*^p < 0.05 and ^***^p < 0.001. Bars represent the SD.

The antifungal activity assay indicated that KA can not only inhibit the formation of sclerotia, but can also inhibit *S. sclerotiorum* mycelial growth. SEM observations revealed that KA was altering cell wall integrity (Figure 3A). KA-treated cells (10 mM KA) showed numerous holes and irregularities in the cell wall that were not observed in the non-treated cells.

In order to understand the metabolic alterations caused by KA in *S. sclerotiorum* cell wall, the mRNA levels of three chitin synthase genes (*CHS1*, *CHS2*, and *CHS3*) and one β-1,3-glucan synthase gene (*GSH*) were measured after KA treatment (10 mM KA; Figure 3B). qRT-PCR analysis revealed that the gene expression of these genes was significantly reduced under KA treatment. The gene expression of *GSH* was 9.3-fold lower in KA-treated cells compared to that detected without KA treatment, while the gene expression of *CHS1*, *CHS2*, and *CHS3* decreased by 73, 85, and 87%, respectively. These results suggested that KA is not only inhibiting melanin biosynthesis but also inhibiting the biosynthesis of chitin and β-1,3-glucans.

To confirm that KA was able to inhibit chitin biosynthesis, the concentration of chitin in the KA-treated cells was measured after 0, 1, 2, and 3 days (Figure 3C). Although chitin content increased steadily in non-treated cells, 10 mM KA significantly decreased chitin content, which is consistent with the mRNA levels of the chitin synthase genes after treatment with KA. The concentration of chitin decreased by 75, 45, and 63% in the KA-treated cells after 1, 2, and 3 days, respectively.

### KA Reduces Oxalic Acid Content

**Figure 4:**
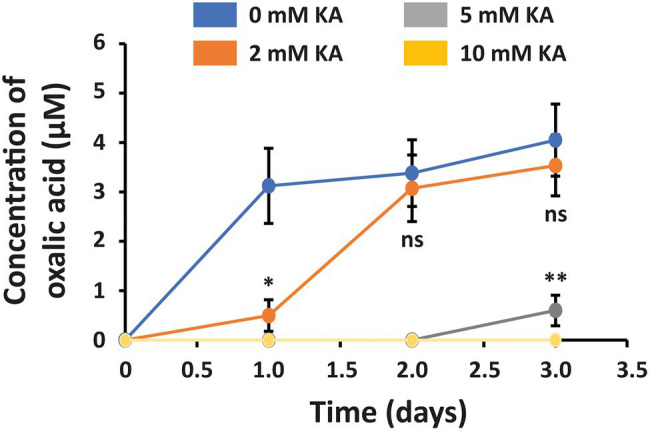
Concentration of oxalic acid in *Sclerotinia sclerotiorum* secretions after treatment with 2, 5, and 10 mM KA (0.28, 0.71, and 1.42 mg/ml KA). The control experiment was carried out in the absence of KA. Each treatment mean represents the average of three repetitions. Significance levels at ^*^p < 0.05 and ^**^p < 0.01. Bars represent the SD.

The concentration of virulence factor oxalic acid when culturing *S. sclerotiorum* in the absence of KA was 3.13 ± 0.76, 3.38 ± 0.67, and 4.05 ± 0.73 μM after 1, 2, and 3 days, respectively (Figure 4). The concentration of oxalic acid was reduced by 12% (no significant difference) and 91% after application of 2 and 5 mM KA, respectively. When treating *S. sclerotiorum* cells with 10 mM KA, no oxalic acid was observed. These results demonstrated that KA is able to inhibit oxalic acid production in *S. sclerotiorum*.

### KA Is Able to Reduce the Symptoms of *Sclerotinia sclerotiorum* in Soybean Pods

**Figure 5:**
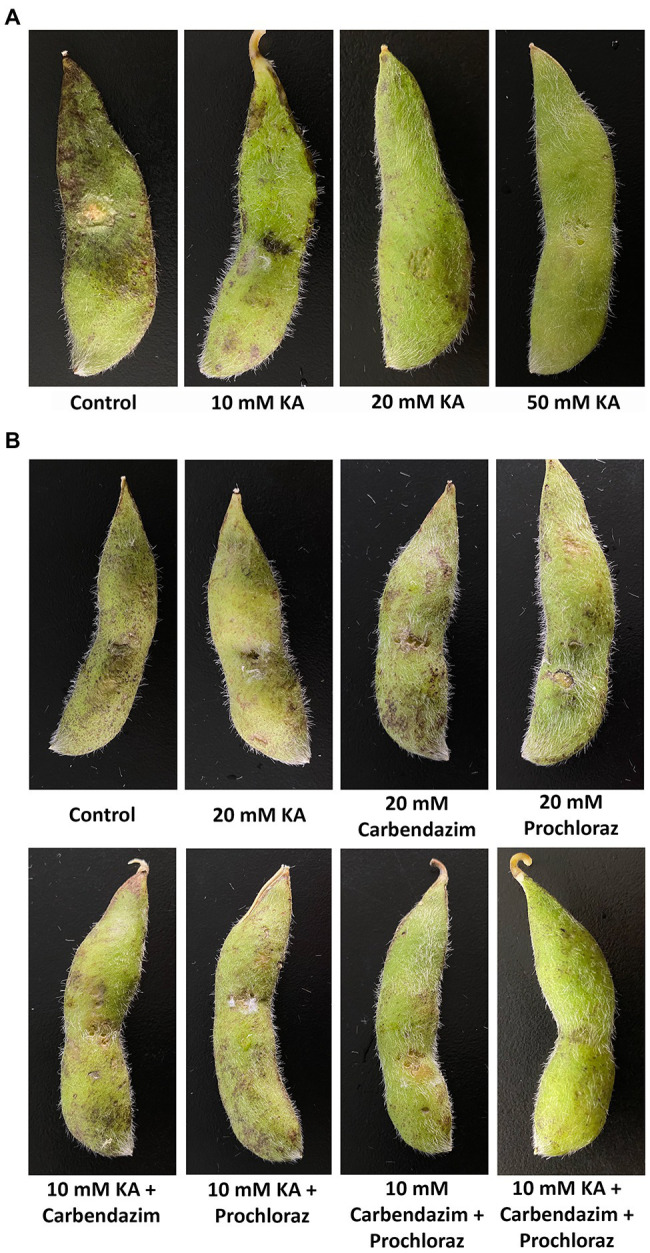
Efficacy of KA and some commercial fungicides for the control of *Sclerotinia sclerotiorum* in soybean pods. **(A)** Preventive applications. **(B)** Curative applications.

Kojic acid showed both curative and preventive abilities, reducing the symptoms of *S. sclerotiorum* in soybean (Figures 5A,B). In preventive applications (Table 1), the lesion length inhibition was proportional to the concentration of KA, observing complete inhibition with 50 mM KA. This, carbendazim and prochloraz at 20 mM provided 64, 65, and 53% lesion length inhibition, respectively, indicating that KA shows similar antifungal activity in preventive applications in comparison with commonly used fungicides.

**Table 1:** Efficacy of kojic acid (KA; MW: 142.1 g/mol) and commercial fungicides carbendazim (MW: 191.2 g/mol), and prochloraz (MW: 376.7 g/mol) for the control of *Sclerotinia sclerotiorum* in soybean pods.

Application	Antifungal agent	Concentration (mM)	Lesion length (mm)^a^	Significance^b^
Preventive	KA	10	4.38 ± 0.58	ns
		20	2.13 ± 0.22	*
		50	0	-
	Carbendazim	20	2.07 ± 0.70	*
		50	0	-
	Prochloraz	20	2.75 ± 0.67	*
		50	0	-
	Control (water)	-	5.88 ± 0.95	-
Curative	KA	20	3.78 ± 0.42	*
		50	1.63 ± 0.27	***
	Carbendazim	20	3.85 ± 0.41	*
		50	1.88 ± 0.16	****
	Prochloraz	20	4.00 ± 0.39	*
		50	1.75 ± 0.17	****
	KA + Carbendazim	10 (KA), 10 (Carbendazim)	1.84 ± 0.28	***
	KA + Prochloraz	10 (KA), 10 (Prochloraz)	2.23 ± 0.82	*
	Carbendazim + Prochloraz	10 (Carbendazim), 10 (Prochloraz)	2.43 ± 0.50	**
	KA + Carbendazim + Prochloraz	10 (KA), 10 (Carbendazim), 10 (Prochloraz)	0.96 ± 0.35	****
	Control (water)	-	4.83 ± 0.14	-

a Lesion length (*n* = 60) ± SD.

b Significance levels: ^*^p < 0.05; ^**^p < 0.01; ^***^p < 0.001; ^****^p < 0.0001; and no significance (ns).

In curative applications, the lesion length inhibition of 20 mM KA was slightly higher than those of carbendazim and prochloraz at the same concentration (KA: 22%; carbendazim: 20%; and prochloraz: 17% lesion length inhibition), and the same trend was also observed when using 50 mM concentrations (KA: 66%; carbendazim: 61%; prochloraz: 64% lesion length inhibition). Comparison between the curative and preventive abilities revealed that the three antifungal agents used in this study showed higher preventive ability in comparison with curative ability on *S. sclerotiorum*. After combining 10 mM KA with 10 mM carbendazim or 10 mM KA with 10 mM prochloraz, the inhibitory effect was higher than that detected with the treatment with single fungicides at 20 mM concentration (KA + carbendazim: 62%; KA + prochloraz: 54% lesion length inhibition). When 10 mM KA was applied together with 10 mM carbendazim and 10 mM prochloraz, the inhibitory effect was enhanced up to 80% lesion length inhibition.

## Discussion

In order to evaluate the antifungal activity of KA, this was screened against four fungal plant pathogens, including *B. dothidea*, *C. brevisporum*, *S. sclerotiorum*, and *V. pyri*. These species were selected for the screening because they were recently isolated by our research group, and suppose a relevant threat in China. *Botryosphaeria dothidea* and *C. brevisporum* were found causing stem canker and anthracnose on soybean, whereas *V. pyri* was isolated from pear trunk (Chen et al., 2020; Shi et al., 2020; Song et al., 2020). The obtained results indicated that KA shows much stronger antifungal activity against *S. sclerotiorum* than against the other strains and, for this reason, the rest of the study was performed using *S. sclerotiorum*. As it is common in the studies using *S. sclerotiorum*, only one strain was isolated and this strain was used in the experiments (Clarkson et al., 2014; Ordonez-Valencia et al., 2015; Ranjan et al., 2019).

Regarding the antifungal activity of KA, this was previously reported to show weak antifungal activity *in vitro* against *Glomerella cingulata* and *Botrytis cinerea* (Wei and Ji, 2016), and to inhibit the growth of some human pathogenic yeasts, including *Candida albicans* (MIC = 4.5 M), *Cryptococcus neoformans* (MIC = 0.56 M), and *Trichophyton rubrum* (MIC = 1.13 M; Chee and Lee, 2003). When comparing the antifungal activity of KA to *S. sclerotiorum* and mentioned yeasts, it can be concluded that *S. sclerotiorum* (MIC = 15 mM) is much more sensitive than other fungal strains, which is in agreement with the antifungal assay carried out in this work. The antifungal activity of KA in combination with H_2_O_2_ was screened against *Penicillium* species and *Aspergillus fumigatus* (Kim et al., 2012; Kim and Chan, 2014). Carbendazim and prochloraz showed higher ability to inhibit mycelial growth than KA. The EC_50_ value obtained for prochloraz in this work was in agreement with the EC_50_ values observed for prochloraz against other *S. sclerotiorum* strains (Zhang et al., 2018).

Microscope observations revealed that the agglutination of the fragmented mycelia formed sclerotia; however, the treatment with KA inhibited sclerotia formation. As in the case of KA, Soylu et al. (2007) reported that essential oils from oregano blocked the formation of sclerotia in *S. sclerotiorum*. SEM observations of *S. sclerotiorum* sclerotia revealed that essential oils from oregano were able to cause important morphological alterations. The sclerotia of *S. sclerotiorum* can show different size and shape (Ordonez-Valencia et al., 2015; Nahar et al., 2020). As indicated in the “Materials and Methods” section, experiments were carried out at 28°C. Although the optima temperatures for mycelial growth and sclerotia germination are between 18 and 22°C, the production of sclerotia, which was the main target in this work, is usually induced at slightly higher temperatures, between 24 and 30°C (Abdullah et al., 2008; Euteneuer et al., 2021).

When evaluating the inhibitory activity of KA at different pH conditions, it was observed that the formation of sclerotia was higher at acidic pH values than at alkaline conditions. In agreement, Xu et al. (2015) indicated that the formation of sclerotia by *S. sclerotiorum* was mainly produced at low pH values. The synthesis of oxalic acid by *S. sclerotiorum* has been reported to be involved in the reduction of environmental pH, facilitating the formation of sclerotia. The treatment of *S. sclerotiorum* with KA inhibited the production of acidifying agent oxalic acid, which is consistent with the observed inhibition of sclerotia formation. In this work, it was found that the formation of sclerotia was reduced by some metal ions, such as nickel(II) and copper(II). As far as we know, this is the first study of the effect of metals on sclerotia formation. The addition of metal ions reduced the inhibitory activity of KA, which can be explained considering the ability of KA to coordinate with metals in solution. This fact suggests that KA should not be combined with metal-based fungicides to achieve a high efficacy.

Kojic acid inhibited the formation of melanin DHN in *S. sclerotiorum*, which is necessary for sclerotia formation. In agreement with our result, Lujan et al. (2016) reported the use of KA, phthalide, and tricyclazole to avoid the pigmentation in *S. sclerotiorum*. As indicated in the “Introduction” section, KA is a copper chelator and is known to inhibit tyrosinase activity in human cells reducing the content of melanin. *Sclerotinia sclerotiorum* also contains a copper-dependent tyrosinase that is known to be involved in melanin formation using L-dopa as an intermediate (Melo et al., 2019). However, *S. sclerotiorum* contains another biosynthetic pathway responsible for the synthesis of DHN melanin, which seems to be more important for the formation of sclerotia than the L-dopa-related pathway (Melo et al., 2019). The DHN melanin pathway does not involve the participation of the tyrosinase but involves the participation of the polyketide synthase PKS12, which gene expression was found to be downregulated after KA treatment, and a copper-dependent laccase. Although the obtained results demonstrated that KA is able to inhibit DHN biosynthesis and sclerotia formation in *S. sclerotiorum*, further studies are necessary in order to understand the target enzymes that are affected by KA. Disruption of *SCD1* or *THR1*, which are involved in melanin biosynthesis in *S. sclerotiorum*, did not change the pathogenicity of the fungus but resulted in slower radial growth, less biomass, wider angled hyphal branches, and impaired sclerotial development (Liang et al., 2018).

In this study, KA was found to inhibit chitin and β-1,3-glucan biosynthesis in *S. sclerotiorum*, altering fungal cell wall. Both structures, chitin and β-1,3-glucans, are known to play an important role in cell wall formation and mycelial growth in *S. sclerotiorum* and have been associated with virulence (Ding et al., 2019). Fungicide prochloraz, which also shows chelating properties, was reported to inhibit chitin biosynthesis in *S. sclerotiorum* (Zhang et al., 2018). The expression of chitin and β-1,3-glucan synthases is known to occur along with the formation of melanin in *S. sclerotiorum*, and this effect must be produced due to chitin and β-1,3-glucans serve as a scaffold to which the melanin granules are cross-linked (Melo et al., 2019). In agreement with that report, the treatment with KA simultaneously downregulated the biosyntheses of melanin, chitin, and β-1,3-glucans, suggesting that there must be some connections between these processes.

As indicated in the “Results” section, the treatment with KA reduced the content of oxalic acid in *S. sclerotiorum* secretions. Oxalic acid is one of the major virulence factors of *S. sclerotiorum* and is involved in programmed cell death (PCD) in plants (Cessna et al., 2000; Williams et al., 2011; Jiao et al., 2021). These results suggest that KA is reducing the pathogenicity of *S. sclerotiorum*, which is in agreement with the ability of KA to reduce the lesions of *S. sclerotiorum in vivo*. Although there are several reports confirming the antifungal activity of KA against plant fungal pathogens *in vitro*, this is the first study on the use of KA for the management of a fungal pathogen *in vivo*.

As mentioned in “KA is able to reduce the symptoms of *S. sclerotiorum* in soybean pods” section, KA showed similar, or slightly higher, efficacy in comparison with commercial fungicides carbendazim and prochloraz. Interestingly, combinations of KA with mentioned commercial fungicides improved the efficacy of the commercial fungicides in curative applications. These results can be explained considering the ability of KA to modify cell wall composition, which may improve the antifungal effects of the commercial fungicides. The mode of action of carbendazim is based on the inhibition of microtubule polymerization in cells by acting with *β*-tubulin, which leads to impaired segregation of chromosomes during cell division (Wang et al., 2020); while prochloraz, which is an imidazole fungicide, can block the biosynthesis of ergosterol in fungus by inhibiting the enzymatic activity of iron(II)-dependent lanosterol 14-α demethylase (Zhang et al., 2018). This study reveals an interesting strategy to enhance the activity of commercial agents for the management of *S. sclerotiorum*, and provides new insights on the use of KA for the control of fungal plant diseases.

## Data Availability Statement

The datasets presented in this study can be found in online repositories. The names of the repository/repositories and accession number(s) can be found in the article/Supplementary Material.

## Author Contributions

PL, X-CS, and S-YW designed the experiments. G-YZ and BW performed the experiments. PL and X-CS analyzed the data. PL, S-YW, and G-YZ drafted the manuscript. All authors contributed to the article and approved the submitted version.

## Funding

This study was supported by the National Natural Science Foundation of China (81803407, 3201101306, and 32172441), the Nantong Applied Research Program (JC2020103), the Social and Livelihood Project of Nantong (MS12020069), and the Large Instruments Open Foundation of Nantong University (KFJN2130 and KFJN2135).

## Conflict of Interest

The authors declare that the research was conducted in the absence of any commercial or financial relationships that could be construed as a potential conflict of interest.

## Publisher’s Note

All claims expressed in this article are solely those of the authors and do not necessarily represent those of their affiliated organizations, or those of the publisher, the editors and the reviewers. Any product that may be evaluated in this article, or claim that may be made by its manufacturer, is not guaranteed or endorsed by the publisher.
