# Supplementary material for: Antifungal Mechanism and Efficacy of Kojic Acid for the Control of Sclerotinia sclerotiorum in Soybean
Source: Front Plant Sci. 2022 Mar 11;13:845698. doi: 10.3389/fpls.2022.845698 (PMC8963468; doi:10.3389/fpls.2022.845698)
Supplement: Supplementary file 1 [file Data_Sheet_1.PDF]

Supplementary Material

Antifungal Mechanism and Efficacy of Kojic Acid for the Control of *Sclerotinia sclerotiorum* in Soybean

Gui-Yang Zhu[1], Xin-Chi Shi[1]*, Su-Yan Wang[1]*, Bo Wang[2] and Pedro Laborda[1]*

[1] School of Life Sciences, Nantong University, Nantong 226019, People's Republic of China

[2] Xuzhou Institute of Agricultural Sciences in Xuhuai District, Sweet Potato Research Institute, Xuzhou 221131, People's Republic of China

*Correspondence:

Pedro Laborda; email: pedro@ntu.edu.cn

Su-Yan Wang; email: wangsuyan@ntu.edu.cn

Xin-Chi Shi; email: shxch0301@ntu.edu.cn

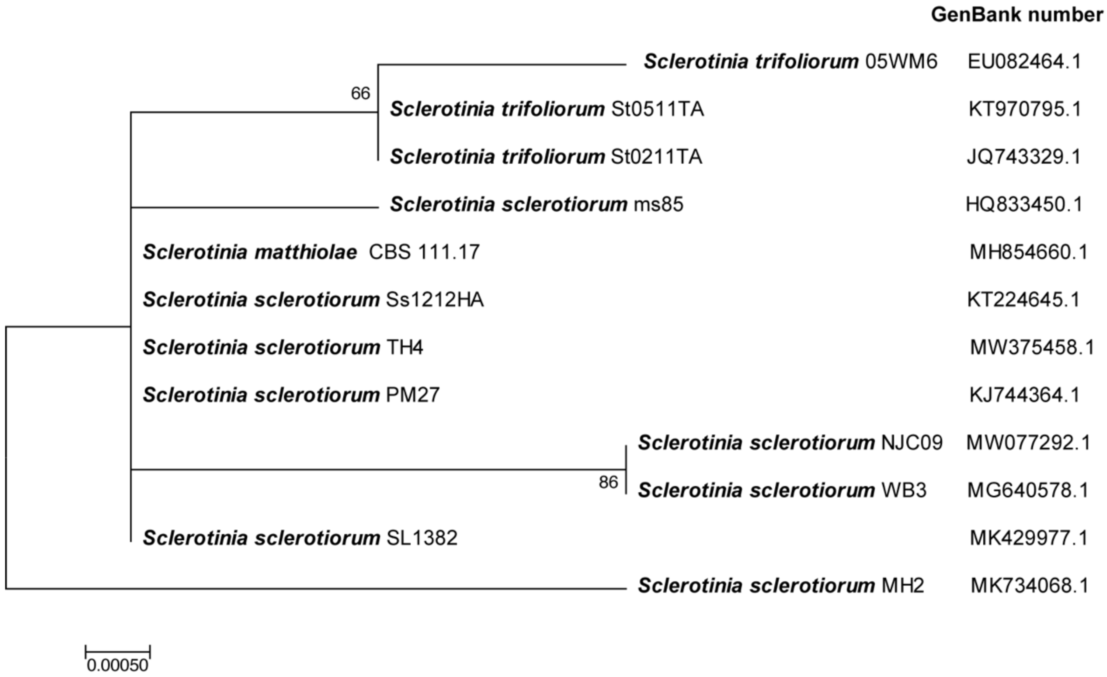

FIGURE S1. Phylogenetic tree of *Sclerotinia sclerotiorum* isolated from soybean plants in Xuzhou, *S. sclerotiorum* strain NJC09.

Small pieces, approximately 0.5 cm^2 in size, of symptomatic tissue were surface sterilized in 1.5% NaOCl for 1 min, and washed twice with sterile H_2O. The pathogen was isolated and cultured on PDA, containing chloramphenicol (50 µg/mL), under darkness at 28 ºC for 3 days. The internal transcribed spacer (*ITS*) was amplified using the standard primers *ITS4* and *ITS5*. The sequence was deposited in GenBank under accession number MW077292. As indicated along the manuscript, the morphology was in agreement with the morphological characteristics of *S. sclerotiorum*, and the isolated strain could cause Sclerotinia rot on soybean pods.

Evolutionary analysis was conducted using MEGA7, with reference strains retrieved from GenBank based on the ITS sequences. The evolutionary history was inferred by using the Maximum Likelihood method based on the Tamura 3-parameter model. The tree with the highest log likelihood (-783.2759) is shown. The percentage of trees in which the associated taxa clustered together is shown next to the branches. Initial trees for the heuristic search were obtained automatically by applying NeighborJoin and

BioNJ algorithms to a matrix of pairwise distances estimated using the Maximum Composite Likelihood approach, and then selecting the topology with superior log likelihood value. The tree is drawn to scale, with branch lengths measured in the number of substitutions per site. All positions containing gaps and missing data were eliminated. There were a total of 522 positions in the final dataset.

Apart from *S. sclerotiorum* NJC09, the analysis involved 11 nucleotide sequences: *S. sclerotiorum* MH2 (Genbank: MK734068; Host: *Physalis pubescens*, Location: China), *S. sclerotiorum* WB3 (GenBank: MG640578; Host: *Galinsoga parviflora*, Location: India), *S. sclerotiorum* SL1382 (GenBank: MK429977; Host: *Fragaria ananassa*, Location: USA), *S. sclerotiorum* PM27 (GenBank: KJ744364; Host: sugar beet, Location: Germany), *S. sclerotiorum* TH4 (GenBank: MW375458; Host: *Panax ginseng*; Location: China), *S. sclerotiorum* Ss1212HA (GenBank: KT224645; Host: *Helychrysum arenarium*; Location: Poland), *S. matthiolae* CBS 111.17 (GenBank: MH854660; Host: not indicated; Location: Switzerland), *S. sclerotiorum* ms85 (GenBank: HQ833450; Host: not indicated; Location: not indicated), *S. trifoliorum* St0211TA (GenBank: JQ743329; Host: *Trifolium ambiguum*; Location: Poland), *S. trifoliorum* St0511TA (GenBank: KT970795; Host: *Trifolium ambiguum*; Location: Poland), and *S. trifoliorum* 05WM6 (GenBank: EU082464; Host: not indicated; Location: not indicated).

TABLE S1. Primers used in qRT-PCR.

Gene	Reference	Forward primer	Reverse primer
PKS12	This work (GenBank number: XM_001585755)	AACTTGCCTCGTTGGCCTGTG	CACCACAACTACCGCAAAGC
PKS13	This work (GenBank number: XM_001586710)	AAATGGGAACGATTGGTGAA	CAGAGTAGAGCCGTGAGTTC
CHS1	(Sousa Melo et al. 2019)	AGATGCGCCATATGGAAGAC	CACAGATGGCTGAGGCTGTA
CHS2	(Sousa Melo et al. 2019)	TGTTGCAGGTCAGAACTTCG	TCCCATGATAGCTCGGAATC
CHS3	(Sousa Melo et al. 2019)	ATGTATCTGGCGGAAGATCG	ACTTACCCGGCACATCAGTC
GSH	(Sousa Melo et al. 2019)	ATCCCGAAGTCGTTCAAATG	TCGGTGTTTTCCATCTCCTC
Actin	(Sousa Melo et al. 2019)	CCCCAGCGTTCTACGTCT	CATGTCAACACGAGCAATG

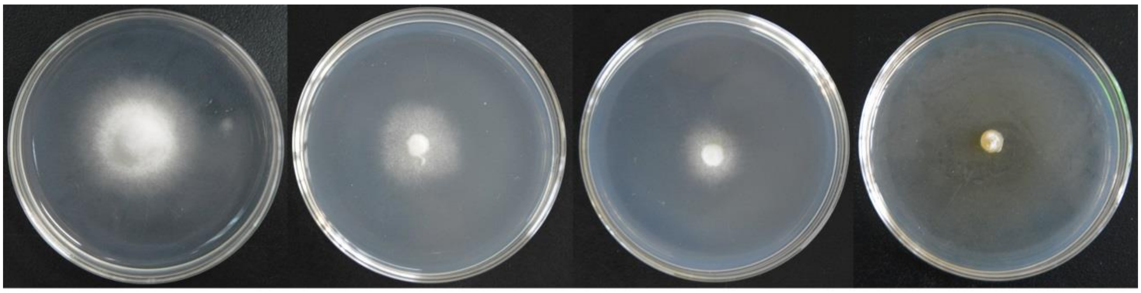

0 mM KA 5 mM KA 10 mM KA 15 mM KA

FIGURE S2. Antifungal activity of 0, 5, 10 and 15 mM kojic acid (KA) against *Sclerotinia sclerotiorum*.

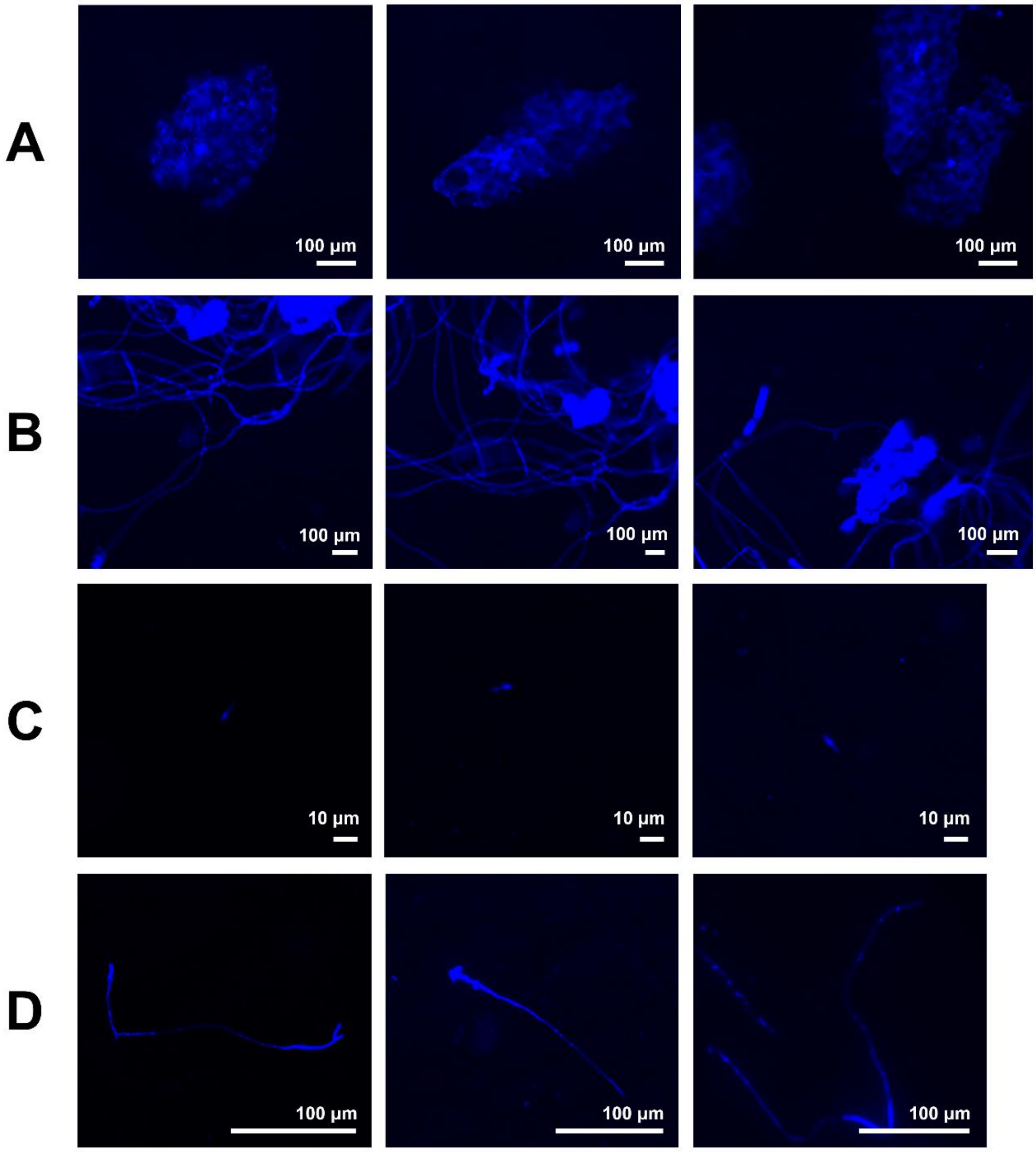

FIGURE S3. Fluorescent live-cell imaging of *Sclerotinia sclerotiorum* cells and antifungal effects of kojic acid (KA). **A** Sclerotia (0 mM KA). **B** Sclerotia with mycelium (0 mM KA). **C** Fragmented mycelia (10 mM KA). **D** Hyphae (10 mM KA).

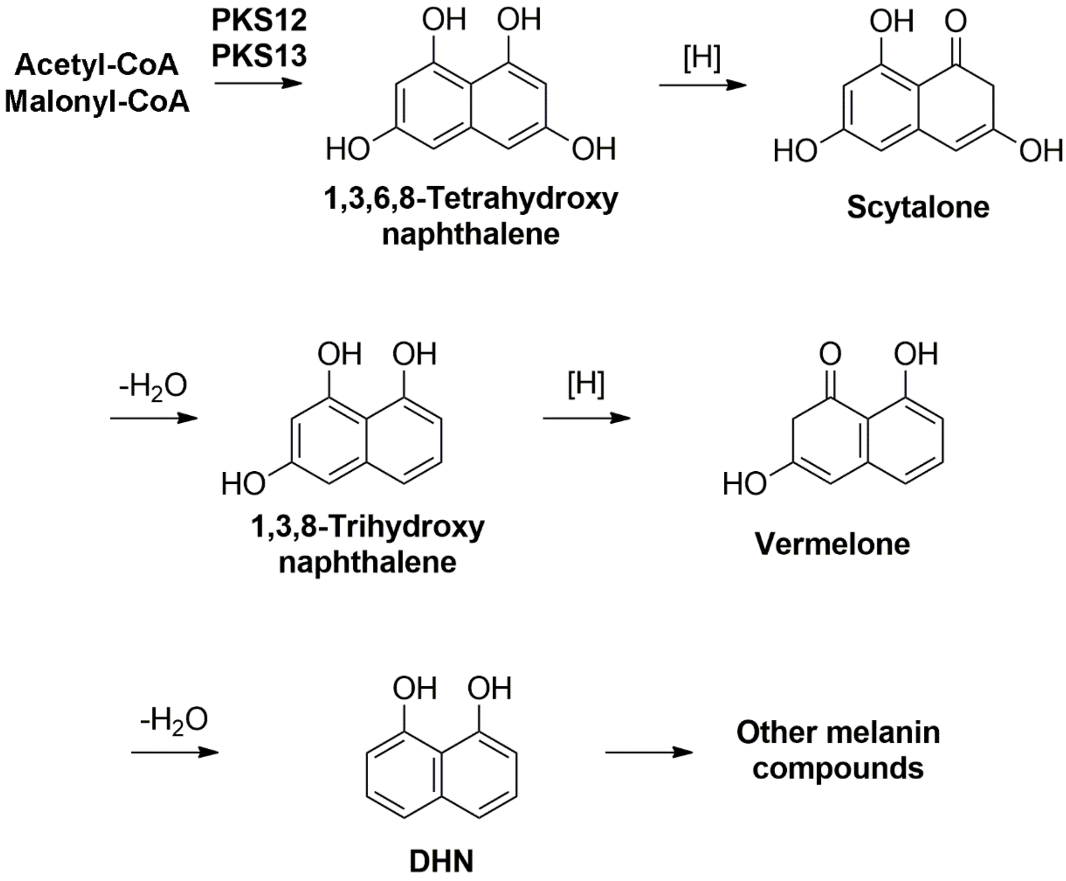

FIGURE S4. Biosynthesis of 1,8-dihydroxynaphthalene (DHN) (Butler et al., 2009).

REFERENCES

Butler, M. J., Gardiner, R. B., and Day, A. W. (2009). Melanin synthesis by *Sclerotinia sclerotiorum*. *Mycologia* 101, 296-304. doi: 10.3852/08-120

Sousa Melo, B., Voltan, A. R., Arruda, W., Cardoso Lopes, F. A., Georg, R. C., and Ulhoa, C. J. (2019). Morphological and molecular aspects of sclerotial development in the phytopathogenic fungus *Sclerotinia sclerotiorum*. *Microbiol. Res.* 229, 126326. doi: 10.1016/j.micres.2019.126326
